# Sleep and circadian rhythm disruption alters the lung transcriptome to predispose to viral infection

**DOI:** 10.1016/j.isci.2022.105877

**Published:** 2022-12-24

**Authors:** Lewis Taylor, Felix Von Lendenfeld, Anna Ashton, Harshmeena Sanghani, Simona Di Pretoro, Laura Usselmann, Maria Veretennikova, Robert Dallmann, Jane A. McKeating, Sridhar Vasudevan, Aarti Jagannath

**Affiliations:** 1 Sleep and Circadian Neuroscience Institute (SCNi), Nuffield Department of Clinical Neurosciences, New Biochemistry Building, University of Oxford, South Parks Road, Oxford OX1 3QU, UK; 2 Department of Pharmacology, University of Oxford, Mansfield Road, Oxford OX1 3QT, UK; 3 Division of Biomedical Sciences, Warwick Medical School, Interdisciplinary Biomedical Research Building, Gibbet Hill Campus, University of Warwick, Coventry CV4 7AL, UK; 4 Zeeman Institute for Systems Biology & Infectious Disease Epidemiology Research, Department of Mathematics, Mathematical Sciences Building, University of Warwick, Coventry CV4 7AL, UK; 5 Nuffield Department of Medicine, University of Oxford, Old Road Campus, Oxford OX3 7BN, UK; 6 Chinese Academy of Medical Sciences (CAMS) Oxford Institute (COI), University of Oxford, Old Road Campus, Oxford OX3 7BN, UK

**Keywords:** Virology, Neuroscience

## Abstract

Sleep and circadian rhythm disruption (SCRD), as encountered during shift work, increases the risk of respiratory viral infection including SARS-CoV-2. However, the mechanism(s) underpinning higher rates of respiratory viral infection following SCRD remain poorly characterized. To address this, we investigated the effects of acute sleep deprivation on the mouse lung transcriptome. Here we show that sleep deprivation profoundly alters the transcriptional landscape of the lung, causing the suppression of both innate and adaptive immune systems, disrupting the circadian clock, and activating genes implicated in SARS-CoV-2 replication, thereby generating a lung environment that could promote viral infection and associated disease pathogenesis. Our study provides a mechanistic explanation of how SCRD increases the risk of respiratory viral infections including SARS-CoV-2 and highlights possible therapeutic avenues for the prevention and treatment of respiratory viral infection.

## Highlights

• Sleep disruption alters the mouse lung transcriptome
• This results in suppressed innate and adaptive immune systems
• The changes are driven by a disrupted circadian clock
• This generates a lung environment that would promote viral infection

## Graphical abstract

**Figure:**
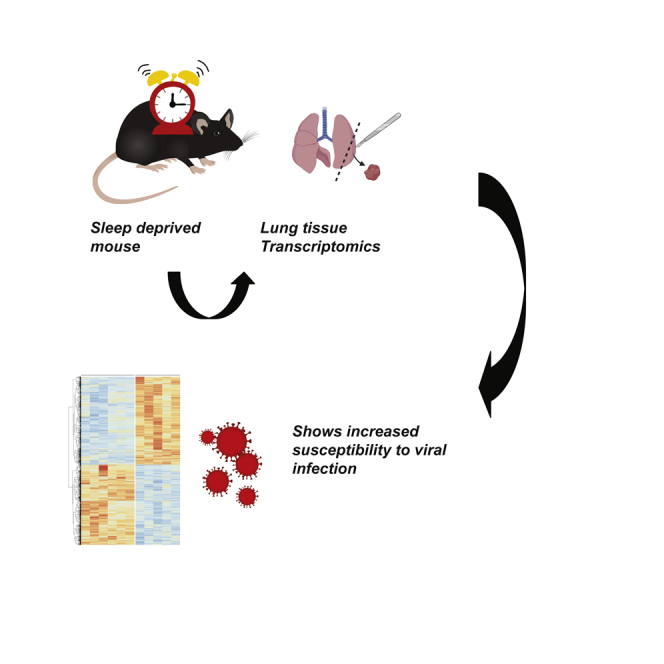

## Introduction

Respiratory viral infections are among the leading causes of mortality worldwide and present a global medical and economic challenge.^1^^,^^2^ Each year, billions of infections lead to millions of deaths, with the annual financial burden estimated at over $100 billion in the US alone.^3^^,^^4^ The recent emergence of severe acute respiratory syndrome coronavirus type 2 (SARS-CoV-2), the causative agent of COVID-19,^5^ has highlighted the impact of respiratory viral infections, with more than 400 million SARS-CoV-2 infections and 5.7 million COVID-19 deaths to date (https://coronavirus.jhu.edu/map.html). An increased understanding of the risk factors and mechanisms driving severe respiratory disease will inform new treatment options.

Sleep and circadian disruption have been reported to cause an increased risk of respiratory infections in mice and humans^6^^,^^7^^,^^8^^,^^9^^,^^10^ and accumulating evidence suggests that shift work and the associated sleep deprivation and circadian rhythm misalignment are risk factors for COVID-19.^11^^,^^12^^,^^13^^,^^14^^,^^15^^,^^16^ Yet, a mechanistic explanation of how sleep and circadian disruption causes higher rates of viral infections remains to be determined.

The immune system is under tight sleep and circadian control. The circadian clock, a molecular transcriptional/translational feedback loop capable of aligning to the external day/night cycle,^17^^,^^18^ generates circadian rhythms; 24-h oscillations in physiology and behavior such as hormone secretion, metabolism, sleep, and immune function.^19^ Indeed, leukocyte trafficking, host-pathogen interaction, and immune cell activation all display diurnal rhythms.^20^ Furthermore, circadian differences in immune responses to vaccination, as well as a diverse range of pathogens and pathogen-derived products are well documented.^21^^,^^22^ Immune responses to Influenza A, Hepatitis A and SARS-CoV-2 vaccines,^23^^,^^24^^,^^25^ and the infectivity of multiple viruses, including Influenza, is dependent on the time of virus challenge.^26^^,^^27^^,^^28^^,^^29^ Disrupting the circadian system in experimental model systems has been reported to increase pro-inflammatory cytokine levels,^30^ and perturb immune cell function and trafficking.^31^ Furthermore, it can promote the replication of a wide range of clinically important viruses including hepatitis B and C, Parainfluenza VirusType 3, Respiratory Syncytial and Influenza A viruses, as shown in transgenic mouse models including BMAL1 KO animals,^6^^,^^27^^,^^32^^,^^33^^,^^34^^,^^35^ emphasizing a central role of the circadian clock in regulating viral infection.^36^

Sleep is one of the most essential circadian regulated behaviors; however, sleep and its homeostasis can be modified and disrupted independently from the circadian clock.^37^^,^^38^ Sleep disruption also leads to immune dysfunction, reducing natural killer cell activity,^39^ modifying pro-inflammatory cytokine production^40^^,^^41^^,^^42^^,^^43^ and blood leukocyte numbers.^44^ Importantly, sleep disruption impairs circadian and immune gene expression in multiple tissues,^45^ including the mouse brain,^46^ liver,^47^ and lung.^48^^,^^49^ A similar disruption of the circadian clock and immune system is seen in blood samples from sleep deprived human subjects.^50^^,^^51^^,^^52^ This dual sleep and circadian rhythm disruption (SCRD) is often encountered by shift workers, particularly those working at night, and is a well-established risk factor for respiratory viral infections. The common cold,^53^ Influenza,^6^^,^^7^^,^^26^ and indeed upper respiratory viral infections in general^8^^,^^10^ are all significantly increased following SCRD. Notably, multiple recent studies have now found an association between shift work, sleep disruption and the risk of developing severe COVID-19. Rizza et al. established a significant association between SARS-CoV-2 infection and night shift work,^54^ and Rowlands et al. found that shift work increases the odds for severe COVID-19 two-fold.^15^ Alongside, Maidstone et al. observed that shift workers, regardless of their occupational group, are more likely to be hospitalized with severe COVID-19; even after adjusting for risk factors such as smoking history, obesity, and asthma.^12^ This is consistent with the finding that people working night shifts irrespective of the job sector are 1.85 times more prone to SARS-CoV-2 infection.^14^ Furthermore, a study on healthcare workers found that each extra hour of sleep reduces the risk for contracting COVID-19 by 12%, whereas workers reporting severe sleeping difficulties experience 88% higher odds of infection.^16^

Despite the increased risk of respiratory viral infections in shift workers, and the established links between sleep, circadian rhythmicity and immune function, the molecular mechanism(s) underpinning higher rates of viral infection following SCRD remain poorly characterized. Therefore, we investigated the effects of acute sleep deprivation on the mouse lung transcriptome and host pathways known to be important for viral lifecycle. In particular we use SARS-CoV-2 as an exemplar, as the recent global research effort has provided a wealth of data detailing the molecular pathways regulating SARS-CoV-2 infectivity and the link between shift work and COVID-19 severity outcomes.^55^^,^^56^^,^^57^^,^^58^ Here we show that 6 h of sleep deprivation in mice profoundly alters the transcriptional architecture of the lung, with a majority of differentially expressed genes associated with host pathways that are essential for viral replication and a suppression of immune and circadian regulated genes with blunted circadian rhythmicity. Moreover, we found that SD causes the differential expression of several host factors implicated in SARS-CoV-2 infection, likely impacting SARS-CoV-2 entry, replication, and trafficking. Together, these data suggest that sleep deprivation alters the lung to provide an environment that could promote respiratory viral infection and pathogenesis.

## Results and discussion

### Acute sleep deprivation alters the lung transcriptome and dampens immune-associated gene expression

**Figure 1:**
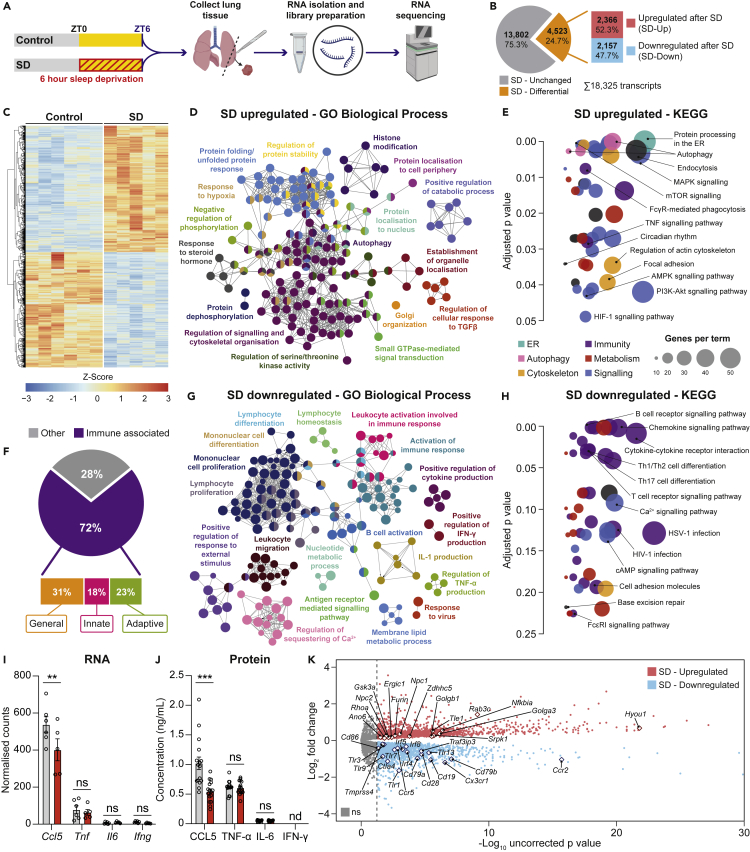
Acute sleep deprivation profoundly alters the lung transcriptome, dampening the immune system and upregulating pathways involved in viral infectivity (A) WT animals were allowed to sleep *ad libitum* (Control) or sleep deprived (SD) between ZT0 – ZT6. Lung tissue was collected at ZT6 and subjected to RNA-sequencing. (B) Of the 18,325 transcripts identified, 4,523 were differentially expressed following SD, with 2,366 upregulated (SD-Up) and 2,157 downregulated (SD-Down). (C) Heatmap of SD differential genes. (D) GO Biological Process (BP) enrichment analysis and term network visualization, and (E) KEGG pathway enrichment analysis of SD upregulated genes. (F) GO BP enrichment analysis and classification of the SD downregulated terms found that 72% were immune associated, and of these 23% were terms involving adaptive immunity, 18% innate immunity and 31% general immunity. (G) GO BP term network visualization, and h KEGG pathway enrichment analysis of SD downregulated genes. GO BP/functional grouping is indicated by color, number of terms/genes is indicated by node size, and edges reflect the relationships between the GO BP terms. (I) Normalized RNA-sequencing counts of *Ccl5*, *Tnf*, *Il6* and *Ifng* (gray – control, red – 6h sleep deprivation). (J) Protein concentration of CCL5, TNF-α, IL-6 and IFN-γ as determined by ELISA (gray – control, red – 6h sleep deprivation). (K) Volcano plot of the significantly differential genes following SD SD-Up (red) and SD-Down (blue) genes are highlighted, with genes in gray being non-significant. For cdata are *Z* score normalized per row and for j and idata are mean ± SEM n = 5–16. Statistical analysis of RNA sequencing data was conducted using DESeq2, and genes with a BH adjusted p value of <0.05 were considered significant. For j and k statistical analysis was conducted by two-way ANOVA with Sidak’s multiple comparisons correction. ns p > 0.05, ∗p < 0.05, ∗∗∗p < 0.001.

To assess the effect of acute sleep deprivation on the lung transcriptome, RNA sequencing (RNA-Seq) was performed on lung tissue isolated from control (*ad libitum* sleep) or 6-h sleep deprived (SD) C57BL/6 mice (Figure 1A). Gene expression analysis identified 2,366 upregulated and 2,157 downregulated transcripts following SD (Figures 1B and 1C and Table S1). We validated our RNA-Seq dataset using qRT-PCR and independent SD lung samples and observed highly correlated results for several top differential genes, confirming the robustness of our transcriptomic analysis (Figure S1). Gene ontology (GO) biological pathway (BP) enrichment analysis of SD upregulated genes showed an enrichment in signal transduction (kinase activity and response to steroid hormones), as well as generic biological processes that are also implicated in viral entry and RNA replication, such as autophagy, Golgi organization, and cellular protein localization (Figure 1D and Table S2). Similar results were observed with Kyoto Encyclopedia of Genes and Genomes (KEGG) pathway analysis of SD upregulated genes, highlighting protein processing in the ER, autophagy, and endocytosis (Figure 1E and Table S2). We also noted an enrichment for circadian rhythm genes (Figure 1E - *Csnk1d*, *Cul1*, *Cry2*, *Csnk1e*, *Clock*, *Rora*, *Arntl*, *Npas2* and *Per1*). Analysis of the SD downregulated genes found that 215 GO BP terms were significantly enriched amongst the SD downregulated genes (adjusted p value<0.01), with 154 (72%) comprising immune system pathways. Of these, innate and adaptive immunity specific terms encompassed 18% and 23% respectively (Figure 1F), suggesting that SD results in widespread immune depression in the lung. Indeed, multiple immune system pathways, including lymphocyte differentiation and proliferation, and leukocyte activation and migration, were repressed following acute SD (Figure 1G and Table S2). KEGG pathway analysis displayed a similar enrichment for immune associated terms in the SD downregulated gene population (Figure 1H and Table S2).

Cytokine production and chemokine signaling were also SD suppressed terms (Figures 1G and 1H). Therefore we measured the levels of the inflammatory mediators TNF-α, IFN-γ, IL-6, and CCL5 in lung homogenates following acute SD to understand how our transcriptome data links to protein production. Alongside the reduced expression of *Ccl5* (Figure 1I), we found a significant reduction in the abundance of CCL5 protein (Figure 1J). Furthermore, multiple chemokine receptors, including *Ccr5* (the cognate receptor for CCL5), *Ccr2*, *Ccr3*, *Ccr6*, *Cxcr3*, *Cxcr5* and *Cx3cr1* were downregulated following SD, strongly suggesting that multiple aspects of chemokine signaling are impacted after SD. To examine how long this suppression of CCL5 persists, we measured lung CCL5 levels in animals allowed 3 h of recovery sleep (RS) after SD. Notably, RS returned CCL5 to baseline levels (Figure S2) and suggests that the immune suppression caused by SD can be reversed once sleep is finally permitted.

In contrast, SD had no impact on *Tnf*, *Il6*, or *Ifng* transcript expression (Figure 1I), which were all much lower than that of Ccl5, and resulted in no significant difference in TNF-α or IL-6 levels, and undetectable amounts of IFN-γ (Figure 1J). Although these results appear to contradict the GO BP analysis that identified the regulation of TNF-α, IFN-γ and cytokine production (Figure 1G), as the baseline level of IL-6 was very low, and IFN-γ undetectable (Figure 1J), any impact on production could only be examined in light of a stimulus that would induce their expression. On the other hand, our data demonstrate that SD would decrease the ability of the immune system to respond to an inflammatory insult, and therefore the full impact of SD on a range of inflammatory processes, including mediator production, will only be unmasked in the face of an immune challenge, such as viral infection. Indeed, *Nfkbia*, a major negative regulator of pro-inflammatory transcription factor nuclear factor kappa (NF-κB), and *Tle1*, another NF-κB repressor, were upregulated after SD (Figure 1K). In addition, GO BP analysis revealed 12 SD upregulated genes implicated in the negative regulation of NF-κB, and 23 SD downregulated genes encoding positive regulators of NF-κB signaling (Table S2); together suggesting that the NF-κB response would be blunted after infection. Similarly, leukocyte migration was also an SD downregulated process. The number of immune cells present in bronchoalveolar lavage fluid is known to be very low under baseline conditions,^59^^,^^60^ but given the marked suppression of chemokine signaling detailed above, a deficit in leukocyte recruitment to the lung in sleep deprived mice would likely occur in response to an infectious insult.

Overall, these results suggest that sleep deprivation alters the lung transcriptome in a manner that would increase susceptibility to SARS-CoV-2 infection, and ideally, this would be confirmed with a study whereby animals are sleep deprived and then exposed to the virus and infectivity assessed. A limitation of this study is such an *in vivo* assessment was not carried out. In the absence of these data, a comparison with existing datasets of infected lungs to assess overlaps in differentially expressed genes presented an intermediary study that would support a future *in vivo* infection study. Therefore, to understand how the SD lung transcriptome compares to SARS-CoV-2 infection, we performed gene set enrichment analysis (GSEA) using the COVID-19 Drug and Gene Set Library^61^ (Table S3). When analyzing the SD upregulated genes, the most significantly enriched gene set was the top 500 genes downregulated in the mouse lung three days post SARS-CoV-2 infection (3 DPI.), as determined by Li et al.^62^ (Figure S3A). Indeed, there was a significant overlap between our SD upregulated genes and the top 500 downregulated genes 3 DPI (Figure S3B - Fisher exact test p value = 7.7 × 10^−26^ and Table S4). However, there was no such enrichment when comparing with the top 500 upregulated genes 3 DPI (Figure S3C). Conversely, when testing the SD downregulated genes, the most enriched gene set was the top 500 genes upregulated in the mouse lung 3 DPI (FiguresS3D and S3E - Fisher exact test p value = 8 × 10^−37^), with no significant overlap with the downregulated 3 DPI (FigureS3F and Table S4). In summary, the transcriptome of the lung following SD is inversely correlated with the lung transcriptome during early-stage SARS-CoV-2 infection. This suggests that SD skews the lung transcriptome away from that needed for mounting an antiviral response, therefore predisposing toward infection. Indeed, *Furin*, which cleaves the SARS-CoV-2 spike protein and regulates particle entry,^63^ was upregulated following SD, whereas several Toll-like receptors (TLRs), which initiate innate immune responses, were all downregulated, including *Tlr3*, *Tlr7*, and *Tlr9* that have been shown to regulate COVID-19 pathogenesis^64^^,^^65^ (Figure 1I). Together, these findings suggest that in the lung, acute SD decreases the ability of the immune system to respond to infection by suppressing both the innate and adaptive immune arms and impacts multiple pathways important for viral host cell entry, intracellular replication, and trafficking.

### Acute sleep deprivation dysregulates the circadian system in the lung

To understand what may be driving these gene expression changes in the lung, we analyzed the transcriptome for the over-representation of transcription factor binding sites in the promoters of genes differentially regulated by SD We found 757 significantly enriched transcription factor signatures (Table S5), with regulators of immediate-early genes (CREB1), and immune-associated genes (CEBPB, NFKB1, TCF7 and STAT3) highly represented (Figure S4). The question as to how much the lung transcriptome changes are actually due to stress should be addressed. Although the SD protocol we used induces relatively less corticosterone than others,^66^^,^^67^ stress and the activation of the HPA axis is unavoidable during SD,^68^ Therefore we analyzed the transcriptome for NR3C1 binding sites in the promoters of genes differentially regulated by sleep deprivation and found that although NR3C1 was over-represented, it was not among the top 20 most over-represented factors which included those specific to immune function such as TCF7, NFKB1 and STAT3, and the circadian clock (CLOCK). Therefore, stress is only a minor contributor toward the SD-induced changes in the lung transcriptome.

**Figure 2:**
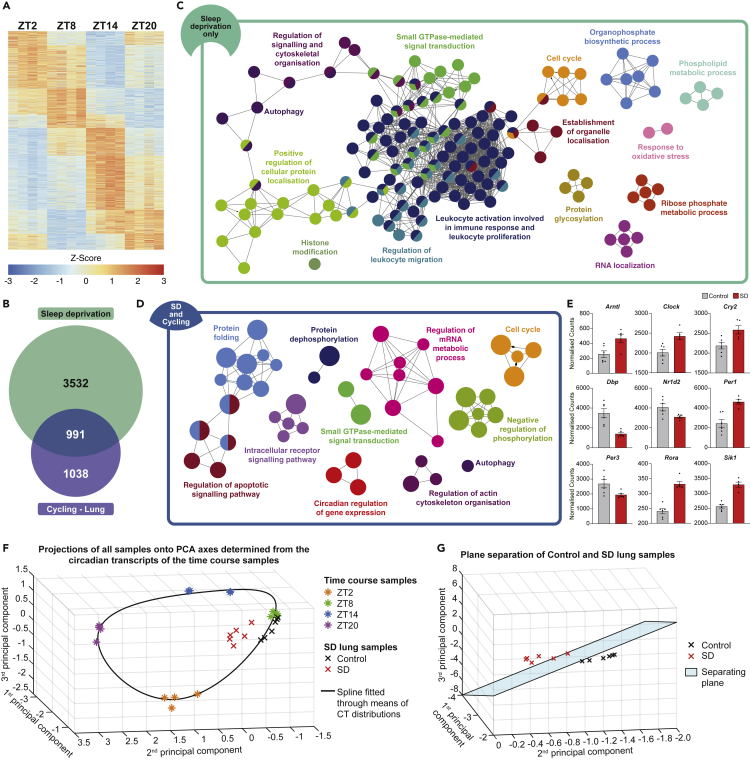
Acute sleep deprivation leads to dysregulation of the circadian system in the lung WT animals were stably entrained to a 12:12 LD cycle, and then placed into constant darkness. Lung samples were then collected at CT2, CT8, CT14 and CT20 and RNA sequencing conducted. (A) Heatmap of the 2,029 significantly cycling genes in the lung. (B) A comparison with genes differentially expressed following SD with the lung circadian genes found 991 rhythmic genes that were also disrupted by SD Network visualization of the significantly enriched GO BP terms of the (C) 3,532 genes that are non-cyclic in the mouse lung, but disrupted by SD (green), and (D) the 991 genes that are cycling in the mouse lung and disrupted by SD (blue). Each node represents a GO BP term. Related terms are grouped by color and edges reflect the relationship between them. (E) RNA sequencing counts of core circadian clock genes in the control and SD lung samples. (F) PCA projection of the circadian (CT) samples in the PC space determined from 10 known circadian transcripts. The black spline represents the estimated circadian behavior of mouse lung under constant conditions, and the graph is oriented such that the separation between the CT samples is as clear as possible. The control samples (black crosses) projected near to the black spline at the approximate expected location, however the SD samples (red crosses) did not project to the same location, demonstrating that SD resulted in circadian disruption in the lung. (G) A Support Vector Machine (SVM) approach with the linear kernel was used to find the plane which optimally separated the control and SD samples in the 3D principal component space. The samples were projected onto the normal of this plane, and a clear separation between the two groups can be seen, which was statistically significant (Wilcoxon rank-sum test – p = 0.0022). Therefore, SD results in circadian disruption of the lung transcriptome. For adata are *Z* score normalized per row and for edata are mean ± SEM n = 5–6. Cycling genes were determined using MetaCycle, and genes with a BH corrected q value of <0.05 were considered as significantly rhythmic. For f and g statistical analysis was conducted using the Wilcoxon rank-sum test.

Notably, our over-representation analysis found that the core circadian transcription factor, CLOCK, was the most significantly enriched (Figure S4). As acute SD has also been previously reported to disrupt circadian rhythmic gene expression in multiple peripheral tissues,^45^^,^^48^^,^^49^^,^^69^ we therefore examined the how sleep deprivation impacts circadian processes in the lung. Rhythmic genes in the mouse lung were identified by sequencing the lung transcriptome at four time points throughout the day separated by 6-h intervals (zeitgeber time(ZT)2, ZT8, ZT14, and ZT20). We identified 2,029 significantly cycling genes in the mouse lung with a 24-h period (JTK q-value <0.05) (Figure 2A and Table S6). Of interest, of these significantly cycling genes, 911 were also disrupted by SD, highlighting that almost 50% of rhythmic genes in the lung are SD sensitive (Figure 2B and Table S4). GO BP enrichment analysis of the 3,532 genes that were non-rhythmic, but SD-differential, revealed immune system associated terms such as leukocyte activation and migration (Figure 2C and Table S2), indicating that many of these immune genes were not circadian regulated, and instead were directly impacted by SD Notably however, GO BP analysis of genes that were both rhythmic and SD-differential showed an enrichment for circadian regulation of gene expression, demonstrating that SD alters the circadian regulatory landscape of the lung (Figure 2D and Table S2). Pathways regulating metabolism, signaling, RNA processing, protein folding, and post-translational protein modification were also rhythmic and dysregulated following SD (Figure 2D), suggesting a widespread disruption of normal circadian lung physiology. Indeed, several circadian transcripts were altered following SD; these included Bmal1 (*Arntl1*), *Clock*, *Per1*, *Cry2*, and *Rora* (Figure 2E), suggesting that at this point of time (ZT6, post SD), the integrity of the core molecular circadian clock, and clock-controlled gene expression, was likely to be compromised.

This disruption to circadian rhythmicity could be examined by time course analysis of gene expression following sleep deprivation; however, all of the information needed to quantify circadian timing is contained in the phase relationships of different rhythmic genes in samples collected at a single time point.^70^^,^^71^ Therefore, we sought to use a bioinformatic approach to quantify the degree of circadian rhythm disruption in the lung caused by acute SD. Principal component analysis of the lung transcriptome allowed us to assess the circadian dysfunction in the lung. The principal directions of a group of 10 circadian transcripts from our ZT transcriptomic dataset (Figure 2A) were used to project all lung samples onto a 3D space and a spline fitted to represent the expected circadian time and behavior of the lung (Figure 2F). Any deviation from this spline would represent an abnormal circadian landscape, and indeed, this is what we found. In contrast to the control samples (black crosses), which fell onto the spline in the expected location, the SD samples (red crosses) were displaced, demonstrating that SD disrupted circadian networks in the lung (Figure 2F). To quantify the impact on the circadian transcriptome, we used a Support Vector Machine approach to locate the plane that maximally separated the control and SD samples. As can be seen in Figure 2G, the optimal plane allowed a clear and significant separation between the SD and control groups (Wilcoxon rank-sum test p = 0.0022; Figure 2G). Overall, these data demonstrate that acute SD alters circadian regulation in the lung, and this disruption could contribute toward the increased susceptibility to respiratory viral infection.

### Host factors implicated in SARS-CoV-2 infection are differentially expressed in the mouse lung following sleep deprivation

**Figure 3:**
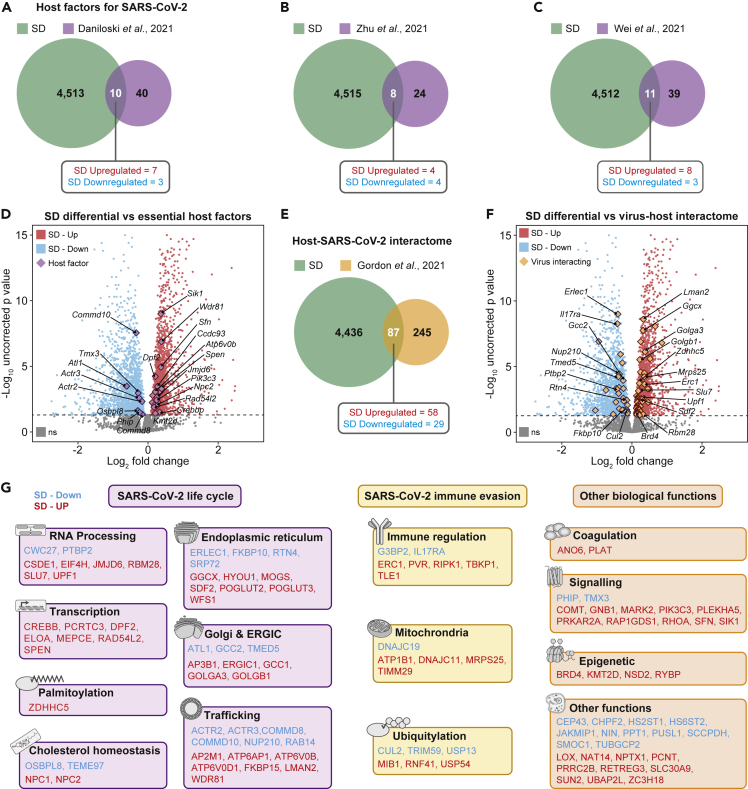
Critical host factors that interact with SARS-CoV-2, and are needed for infection, are differentially expressed in the mouse lung after sleep deprivation (A–C) Venn diagrams of the overlap between all SD differential genes in the mouse lung and critical host factors for viral infection as determined by a Daniloski et al. (2021),^56^ b Zhu et al. (2021),^58^ and c Wei et al. (2021).^57^ (D) Volcano plot of significant SD differential genes (Up – red, Down – blue and non-significant after BH p value correction – gray) with overlapping critical host factors highlighted (purple diamonds) and a subset labeled. (E) The intersection between SD differential genes in the mouse lung and the SARS-CoV-2-human protein interactome as determined by Gordon et al. (2020b).^55^ (F) Volcano plot of SD differential genes with overlapping SARS-CoV-2-human protein interactors highlighted (yellow diamonds) and a subset labeled. (G) Functional classification of the critical host factors and the SARS-CoV-2-human protein interactors that were found to be differentially expressed in the mouse lung following SD Boxes are colored according to the functional role in viral infectivity. Statistical significance of the overlap between SD differential genes and SARS-CoV-2 host factors and host interactome was assessed by two-tailed Fisher exact test. n = 5–6. ERGIC = endoplasmic reticulum-Golgi apparatus intermediate compartment.

Our data shows that acute SD modifies the transcriptional landscape of the lung in two keys ways to promote infection by respiratory viruses, Firstly, by suppressing the innate and adaptive immune responses, and secondly by disrupting the normal circadian regulatory landscape and physiology of the lung. Three independent studies by Daniloski et al.,^56^ Zhu et al.,^58^ and Wei et al.^57^ conducted genome-wide CRISPR loss-of-function screens to identify genes regulating SARS-CoV-2 infection. Therefore, we used these data to examine whether SD changes the expression of host factors required by SARS-CoV-2. Daniloski et al., using human alveolar epithelial cells, identified 1,200 potentially relevant genes for SARS-CoV-2 replication and investigated the 50 most highly enriched.^56^ Of the 50, 10 were dysregulated following SD (Figures 3A and 3D - ACTR2, ACTR3, ATL1, ATP6AP1, ATP6V0B, ATP6V0D1, PIK3C3, SFN, SPEN and WDR81 and Table S4), of which 8 could be assigned a putative function in SARS-CoV-2 replication (Figure 3G). For example, members of the vacuolar-ATPase proton pump, (ATP6V0B, ATP6AP1, and ATP6V0D1), implicated in activation of the SARS-CoV-2 spike protein that is required for viral entry, and ACTR2 and ACTR3, part of the ARP2/3 complex, which functions in endosomal trafficking pathways. Zhu et al. identified 32 genes with a potential role in viral entry,^58^ of which 8 were SD-differential genes (Figures 3B and 3D and Table S4); four each being up- (NPC1, NPC2, CCDC93, WDR81) and downregulated (COMMD8, COMMD10, ACTR2, ACTR3). All 8 genes play a role in endosomal entry, endolysosomal fusion, or endosome recycling (Figure 3G). Cross-referencing our SD-differential genes to the 50 most enriched host factors identified by Wei et al.^57^ revealed an intersection of 11 genes (Figure 3C and Table S4), 8 of which were upregulated and associated primarily with transcriptional regulation (DPF2, JMJD6, RAD54L2, CREBBP, RYBP, ELOA, KMT2D, SIK1 – Figure 3G). The effect of SD on the individual transcripts that encode for these putative SARS-CoV-2 host factors across all three studies is illustrated in Figure 3D. Taken together therefore, acute SD clearly amplifies many host factors and processes that influence multiple steps in the SARS-CoV-2 life cycle.

We next explored if SD-differential genes encode host proteins known to physically interact with SARS-CoV-2 encoded proteins. Gordon et al. interrogated human host factors that interact with 26 of the 29 SARS-CoV-2 proteins.^55^ The authors identified 332 high confidence human-virus protein-protein interactions, of which 87 overlapped with our SD-differential genes (Figures 3E and 3F and Table S4). Of interest, at least 40 of the overlapping genes have a putative function in viral replication, such as RNA processing, ER protein quality control, or intracellular trafficking (Figure 3G). Furthermore, 18 of the overlapping host factors are involved in mitochondrial processes, ubiquitination, or immune regulation, that may function in SARS-CoV-2 immune evasion (Figure 3G). Alongside, regulators of signaling pathways, coagulation, and epigenetic modifiers represent some of the other dysregulated classes of interactors that likely impact SARS-CoV-2 infection. Overall, these findings demonstrate that SD causes the differential expression of several host factors that interact with, and are implicated in, SARS-CoV-2 infection that may potentiate virus replication.

### The effect of sleep deprivation on SARS-CoV-2 life cycle genes

**Figure 4:**
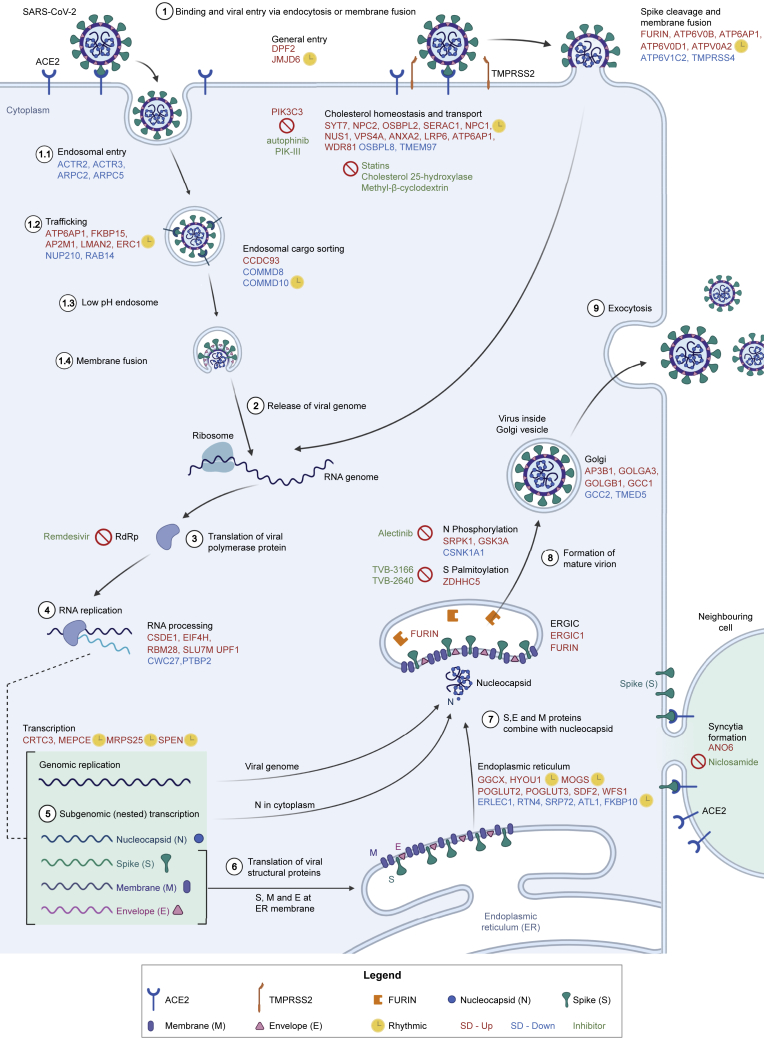
Involvement of differentially expressed genes after sleep deprivation in the SARS-CoV-2 life cycle (1) SARS-CoV-2 binds ACE2 and enters via endocytosis or membrane fusion, depending on the availability of TMPRSS2/4. (2) The viral RNA genome is released into the cytoplasm and (3–4) replicated and (5) transcribed by RdRp. (6) Viral structural proteins are translated by host ribosomes. (7–8) The virion assembles and (9) is released. All differentially expressed genes shown (red font for SD-Up, blue font for SD-Down) apart from *FURIN*, *TMPRSS4*, *GSK3A*, *SRPK1*, and *CSNK1A1* are critical host factors overlapping with at least one of the studies from Gordon et al. (2020b),^55^ Daniloski et al. (2021),^56^ Wei et al. (2021),^57^ or Zhu et al. (2021).^58^ Drugs targeting SD differential or viral genes mentioned are in green font. Cycling genes are denoted by a yellow clock. ACE2 = angiotensin-converting enzyme 2, ERGIC = ER-Golgi apparatus intermediate compartment, RdRp = RNA-dependent RNA polymerase, TMPRSS2 = transmembrane protease serine 2. Adapted from Du et al. (2009) and from “Coronavirus Replication Cycle” by BioRender.com. Created with BioRender.com.

When taken together, our data suggest that acute SD impacts many host processes important for the viral life cycle. Using SARS-CoV-2 as an exemplar, we propose a mechanistic pathway, synthesized from the data presented above, by which the SD-differential genes facilitate viral entry, replication, and trafficking (Figure 4). The extracellular transmembrane protease serine 4 (*Tmprss4*), the protease *Furin*, and *Atp6v0b*, *Atp6ap1*, and *Atp6v0d1* (members of the vacuolar-ATPase proton pump) all contribute toward spike protein activation and cleavage,^72^^,^^73^ and were differentially expressed following SD, suggesting an increase in virus entry. Following intracellular capsid uncoating the viral RNA is replicated within double membrane vesicles, translated by host ribosomes, and new virus particles assembled and trafficked via the Golgi/ER pathway for release by exocytosis. All these pathways were dysregulated by SD, including transcriptional modulation, endolysosomal fusion, endosome recycling (Figure 4).

Thirteen genes implicated in intracellular cholesterol trafficking (*Tmem97*, *Syt7*, *Npc1*, *Npc2*, *Osbpl2*, *Serac1*, *Nus1*, *Vps4a*, *Anxa2*, *Lrp6*, *Atp6ap1*, *Pik3c3*, and *Wdr81*) were differentially expressed following SD, in line with previous findings showing SD driven disruption of cholesterol metabolism.^74^ This was of interest to us, as three of the four cross-referenced SARS-CoV-2 host factor studies (Figure 3) identified disrupted cholesterol homeostasis as a risk factor for infection. Plasma membrane cholesterol is required for SARS-CoV-2 fusion and cell entry,^75^ a pathway common to most enveloped viruses. Furthermore, statins have been found to reduce recovery time and decrease the risk for COVID-19 morbidity and mortality.^76^^,^^77^ How cholesterol impacts SARS-CoV-2 pathogenesis is currently unclear; however, lipid raft disruption, modification of membrane biophysics, alteration of viral stability and maturation, and immune dysfunction have all been suggested as potential mechanisms.^78^^,^^79^^,^^80^

Finally, SD alters post-translational protein modification that regulates multiple aspects of SARS-CoV-2 replication. For example, the viral nucleocapsid protein is phosphorylated by SRPK1, GSK-3α, and CSNK1^81^ and genes encoding all three kinases were differentially expressed in the lungs after SD. Palmitoylation of the Spike envelope glycoprotein is necessary for infectivity. Knockdown of ZDHHC5, a palmitoyltransferase, resulted in spike protein depalmitoylation and compromised membrane fusion and viral entry,^82^ and SD resulted in increased *Zdhhc5* transcripts in the lung. Overall, these findings suggest that SD could promote SARS-CoV-2 replication by dysregulating many genes involved in its life cycle.

### The effect of sleep deprivation on the anti-SARS-CoV-2 immune response and viral immune evasion

**Figure 5:**
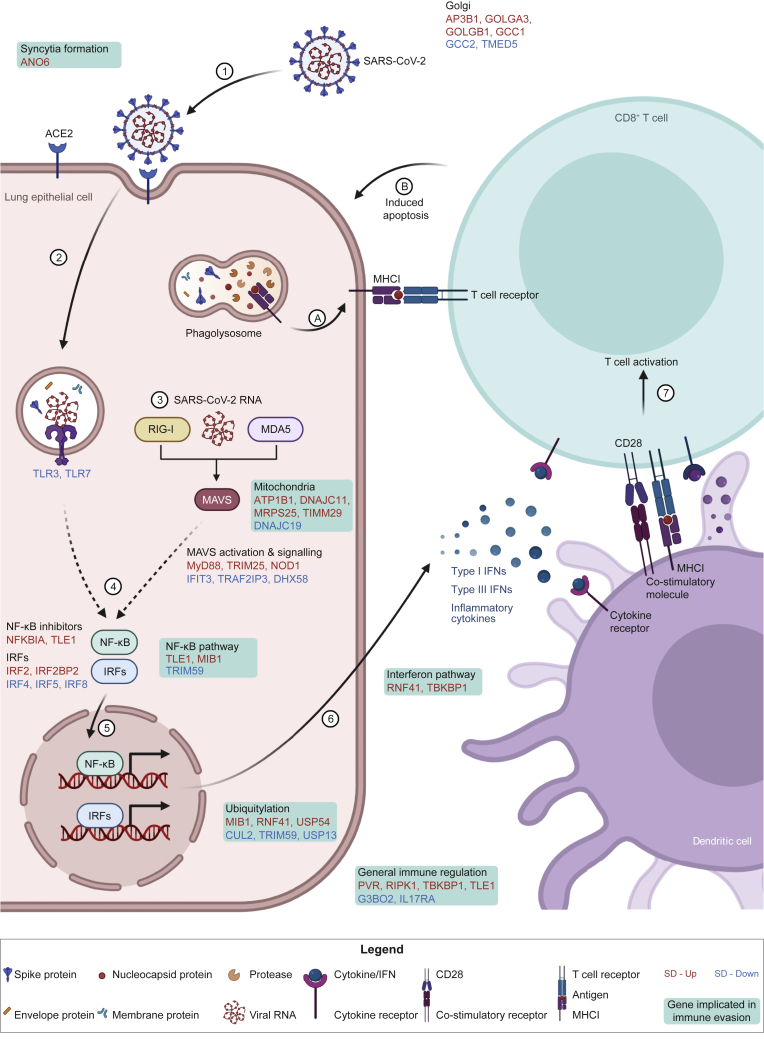
The effect of sleep deprivation on the anti-SARS-CoV-2 immune response and viral immune evasion (1) The virus enters the host cell. Viral RNA is detected by (2) endosomal TLRs or (3) cytosolic RIG-I and MDA5, which activate MAVS. (4) Both recognition events activate NF-κB and IRFs, which (5) translocate into the nucleus to (6) drive the expression of IFNs and inflammatory cytokines to amplify the antiviral immune program, for example by priming dendritic cells to sample and display viral antigens to (7) activate naive CD8^+^T cells. Inside the infected host cell, (A) viral material is broken down and displayed on the cell surface by MHC class I molecules. If the antigen is recognised by CD8^+^T cells (B) it induces apoptosis of the infected host cell. All differentially expressed genes shown (red font for SD-Up, blue font for SD-Down) are involved in the acute innate immune response against SARS-CoV-2. Genes in green-shaded text boxes are implicated in viral immune evasion, as described in Gordon et al., (2020b).^55^ IFN = interferon, IRF = interferon regulatory factor, MAVS = mitochondrial antiviral signaling protein, MDA5 = melanoma differentiation-associated protein 5, MHC I = major histocompatibility complex molecule class I, NF-κB = nuclear factor kappa B, RIG-I = retinoic acid-inducible gene 1. Adapted from “Acute Immune Responses to Coronaviruses”, by BioRender.com. Created with BioRender.com.

Alongside the impact on viral replication, our data shows that SD can suppress immune associated genes allowing viral persistence. Analysis of the SD lung transcriptome shows altered regulation of several components of the immune system (Figure 5). The regulators of interferon production, RNF41 and TBKBP1, are targeted by SARS-CoV-2 proteins^55^ and their genes were differentially expressed following SD. Furthermore, SD caused the differential expression of the E3 ubiquitin ligases *Mib1* and *Trim59*, which induce and repress NF-κB, respectively,^83^^,^^84^ alongside the NF-κB repressor, *Tle1*. These proteins have been shown to associate with SARS-CoV-2 proteins, suggesting that infection interferes with the NF-κB pathway as an immune evasion strategy. Accumulating evidence suggests that SARS-CoV-2 exploits the host ubiquitination machinery to evade the innate immune response,^85^^,^^86^ and intriguingly, six SD differential genes (*Mib1*, *Rnf41*, *Usp54*, *Cul2*, Trim59, Usp13) functionally implicated in ubiquitination, encode proteins that interact with SARS-CoV-2 proteins.^55^ Severe COVID-19 is sometimes associated with syncytia in the lung; multinucleated single cells formed by the fusion of SARS-CoV-2 infected cells to allow viral genome transfer without activating the immune system.^87^ Recently, ANO6 has been found to regulate syncytia formation,^88^ and, of interest, we found that *Ano6* was upregulated following SD Finally, the manipulation of multiple immune-linked mitochondrial functions is another approach by which respiratory viruses including coronaviruses evade the host immune system,^89^ and notably we found five SD differential mitochondrial host genes (*Dnajc19*, *Atp1b1*, *Dnajc11*, *Mrps25*, and *Timm29*) which are known to engage in SARS-CoV-2 protein-protein interactions.^55^ Taken together therefore, this highlights how acute SD may specifically promote viral immune evasion via multiple complementary pathways.

In conclusion, this study shows that SD alters the transcriptomic landscape in the mouse lung in a manner that could explain the increased risk of respiratory viral infections, as well as severe COVID-19, associated with SCRD and shift work. Suppression of the immune response and promotion of SARS-CoV-2 replication and immune evasion are among the most relevant pathways deregulated by SD. Furthermore, we found a widespread disruption of circadian rhythmicity in the lung following sleep deprivation, which could precipitate and/or exacerbate the negative consequences of SCRD.

### Limitations of the study

One limitation of this study is that it only assessed the effect of acute sleep deprivation, not chronic, which would also be very informative. Another limitation of this study was that an *in vivo* challenge experiment was not undertaken. The hypotheses proposed in this study require validation by challenging mice with SARS-CoV-2 after SD; indeed, this would be an important follow-up study. However, these findings help explain why SCRD is associated with severe COVID-19 and could guide future efforts toward understanding the mechanisms underlying SARS-CoV-2 pathogenesis. Importantly, our observations are applicable to a wide range of respiratory viruses and may inform avenues to develop new therapeutic efforts.

## STAR★Methods

### Key resources table

**Table:** 

REAGENT or RESOURCE	SOURCE	IDENTIFIER
**Critical commercial assays**		

Illumina TruSeq Stranded Total RNA library prep gold kit	Illumina	Cat#20020598
NextSeq 550 and a Nextseq 500/500 v2.5 75 cycle kit	Illumina	Cat#20024906
KAPA library quantification kit	Roche	Cat#07960140001
BCA protein assay kit	Life Technologies	Cat#23225
Mouse TNF-alpha DuoSet ELISA	R&D Systems	DY410-05
Mouse IL-6 DuoSet ELISA	R&D Systems	DY406
Mouse IFN-gamma DuoSet ELISA	R&D Systems	DY485
Mouse CCL5/RANTES DuoSet ELISA	R&D Systems	DY478

**Deposited data**		

Chea3 transcription factor database	https://maayanlab.cloud/Enrichr/	
COVID-19 Drug and Gene Set Library	https://maayanlab.cloud/Enrichr/	
Fastq files	NCBI SRA	Accession number PRJNA914246

**Experimental models: Organisms/Strains**		

Mouse: C56Bl6/J	Envigo	

**Oligonucleotides**		

Primer sequences in Table S7		

**Software and algorithms**		

Clocklab	Actimetrics	https://www.actimetrics.com/products/clocklab/
Prism 8	GraphPad	https://www.graphpad.com/
HISAT2	(Kim et al., 2019)^90^	http://daehwankimlab.github.io/hisat2/about/
FeatureCounts	(Liao et al., 2014)^91^	http://subread.sourceforge.net/
DeSeq2	(Love et al., 2014)^92^	https://bioconductor.org/packages/release/bioc/html/DESeq2.html

### Resource availability

#### Lead contact

Further information and requests for resources and reagents should be directed to and will be fulfilled by the lead contact, Aarti Jagannath (aarti.jagannath@ndcn.ox.ac.uk).

#### Materials availability

This study did not generate new unique reagents.

### Experimental model and subject details

#### Animals

All studies were conducted using male C57BL/6 mice over 8 weeks of age and, unless otherwise indicated, animals were group housed with *ad libitum* access to food and water under a 12:12 h light/dark cycle (100 lux from white LED lamps). All animal procedures were conducted in accordance with the UK Home Office regulations (Guidance on the Operation of Animals(Scientific Procedures Act) 1986) and the University of Oxford’s Policy on the Use of Animals in Scientific research, following the principles of the 3Rs. For circadian time course analysis, lung tissue was collected at zeitgeber time (ZT)2, ZT8, ZT14, and ZT20. The premise of this study was that COVID-19 infection outcomes are worse in shift-workers. Sleep deprivation (SD) is a core feature of shift work, and the effects of acute SD on both brain and peripheral transcriptomes in mice has been shown to replicate the changes seen in humans shift work-like paradigms, where humans are more chronically sleep-deprived.^50^^,^^93^ Thus, we sought to profile the effects of acute SD on the lung transcriptome. For the SD experiments, animals were kept awake for 6 h between ZT0 and ZT6 by providing novel objects to elicit exploratory behaviour, as previously described.^94^ The animals were then sacrificed, and lung tissue collected. Control animals were allowed to sleep *ad libitum* between ZT0 and ZT6. Recovery sleep (RS) animals were sleep deprived for 6 h, as detailed above, and then allowed to sleep *ad libitum* for 3 h before being sacrificed and lung tissue collected.

### Method details

#### RNA extraction and RNA sequencing library preparation

Total RNA from lung tissue samples was extracted using TRIzol and the RNeasy Mini Kit (Qiagen). Lung tissue was mechanically disrupted in 700 μL of TRIzol and 140 μL of chloroform was added and the sample thoroughly mixed. Following a 3 min incubation at RT, the sample was then centrifuged for 15minat 15,000 xg, 4°C. The clear top layer was then carefully collected, mixed with an equal volume of 70% ethanol and RNA extracted using the RNeasy Mini Kit, with on-column DNase digestion, following the manufacturer’s instructions. RNA was eluted in water and RNA concentration and quality were measured using a TapeStation system (Agilent) with the High Sensitivity RNA ScreenTape assay. mRNA purification and cDNA synthesis for the sequencing library were performed according to the Illumina Stranded mRNA Prep protocol (20040534) using the following index kit: IDT for Illumina RNA UD Indexes Set A, Ligation (20040553). Quality and concentration of the final libraries were checked with the KAPA Library Quantification Kit (Roche Diagnostics) in a StepOnePlus thermal cycler (Applied Biosystems) according to manufacturer’s instructions. All cDNA libraries were sequenced using a paired-end strategy (read length 150 bp) on an Illumina NovaSeq platform.

#### Lung protein extraction

Lung tissue was placed into an appropriate volume of tissue lysis buffer (500 μL/10 mg tissue – 100 mM Tris, 150 mM NaCl, 1 mM EGTA, 1 mM EDTA, 1% Triton-X100, 0.5% Sodium deoxycholate, pH 7.4) supplemented with protease inhibitors (Roche, UK), and then lysed in a glass dounce homogeniser (Sigma, UK). The samples were incubated on ice for 10 min, vortexed, and then placed back on ice for a further 10 min before being centrifuged for 20minat 13,000 xg, 4°C. The protein concentration of the debris free supernatant was determined using the Pierce™ BCA Protein Assay Kit (Thermo Fisher scientific, Loughborough, UK) following the manufacturer’s protocol. The samples were then diluted to 1 mg/mL using reagent diluent (RD – PBS + 1% BSA), aliquoted and then stored at −80°C.

#### ELISA

The concentration of murine CCL5, TNF-α, IL-6 and IFN-γ in total lung homogenate was determined using DuoSet® sandwich ELISA assays (R & D systems). To begin, a 96 well MAXISORP plate (Thermo Scientific) was coated with capture antibody, diluted to the desired working concentration in PBS, overnight at RT. The plate was then washed by completely filling each well with wash buffer (PBS containing 0.05% tween), followed by aspiration, four times. Plates were then blocked by the addition of 300 μL of RD per well and incubation for 1 h at RT. The plates were then washed four times with wash buffer and 100 μL of sample or protein standard diluted in reagent diluent was added per well and the plate incubated for 2 h at RT. Following another wash step, detection antibody diluted in reagent diluent was added to each well and the plate incubated for 2 h at RT. The plates were subjected to another wash step and streptavidin-HRP added to each well and the plate incubated for 20minat RT. The plates then had one final round of washing after which 55 μL of 1-Step Ultra TMB-ELISA solution was added to each well and the plates incubated for 15minat RT in the dark. Finally, 55 μL of 2N H_2_SO_4_ was added to each well to stop the HRP reaction and the absorbance at 450 nm for each well was determined using a FLUOstar OMEGA plate reader. These values were corrected by subtracting absorbance at 570 nm. The amount of each analyte was then determined by interpolation from the protein standard curve, taking into account the dilution factor of each sample.

#### qRT-PCR

Total RNA was extracted from mouse lung tissue as detailed above and cDNA was synthesized using the qScript cDNA Synthesis Kit (Quantabio). mRNA was quantified using the QuantiFast SYBR Green PCR Kit (Qiagen) in a StepOnePlus thermal cycler. Cycling conditions were 95 °C for 5 min, and 40 cycles of 95 °C for 10 s, 60 °C for 30 s, 72 °C for 12 s. The cycle thresholds for each gene were normalized using ActB, Gapdh, and Rn18s as housekeeping genes following the 2^ˆ−ΔCt^ method. The primers used qRT-PCR analysis are listed in Table S7.

#### Processing of RNA sequencing data

Raw RNA-Seq data processing (quality control, trimming, mapping to the genome, and read counting) was performed using tools embedded in Galaxy (v21.05).^95^ The fastqsanger files containing the raw sequencing data were uploaded to the public Galaxy server at usegalaxy.org. FastQC (v0.11.8) (https://www.bioinformatics.babraham.ac.uk/projects/fastqc/) was used for quality control of sequencing data. For quality and adapter trimming, Trim Galore! (v0.6.3) (https://www.bioinformatics.babraham.ac.uk/projects/trim_galore/) was employed to remove low-quality bases, short reads, and Illumina adapters. Nextera transposase was specified as the adapter sequence to be trimmed and Trim Galore! was instructed to remove 1 bp from the 5’ end of both read 1 and 2. FastQC was rerun to assess the quality improvement. High quality reads were then mapped to the *Mus musculus* (mm10) reference genome using HISAT2 (v2.1.0),^90^ specifying the strand information as reverse. featureCounts (v2.0.1)^91^ was run to quantify the number of reads mapped to each gene. The featureCounts built-in mm10 gene annotation file was selected and under paired-end reads options, the option to count fragments instead of reads was enabled. The generated counts files were converted to CSV and downloaded for downstream differential gene expression analysis in R. MultiQC (v1.9)^96^ was used to aggregate FastQC, HISAT2, and featureCounts results.

#### Differential gene expression analysis

To identify differentially expressed genes in the SD and times series (ZT) datasets, the DESeq2 package (v1.32.0)^92^ was used in R (v4.1.0). DESeq2 corrects for multiple testing using the Benjamini-Hochberg (BH) method, and only genes with a BH adjusted p value <0.05 were considered statistically significant. Heatmaps were drawn using the pheatmap function from the pheatmap package (v1.0.12). Volcano plots were generated using the ggplot2 package (v.3.3.5).

To detect periodicity in the time series (ZT) data, the MetaCycle R package (v1.2.0) was used.^97^ The meta2d function was run using the MetaCycle web application (MetaCycleApp) based on the shiny package (v1.6.0). The following parameters were specified: minper = 24, maxper = 24, ARSdefaultPER = 24, cycMethod = JTK, combinePvalue = fisher. Any gene with a corrected q value of <0.05 was considered significantly rhythmic. The MetaCycleApp was downloaded from https://github.com/gangwug/MetaCycleApp.

#### Functional enrichment analysis

Functional enrichment analysis of SD-associated genes and cycling genes was conducted using the clusterProfiler R package (v4.0.0).^98^ GO BP and KEGG analysis was performed using the enrichGO function, with org.Mm.eg.db (v3.13.0) as the *Mus musculus* genome annotation (GO BP parameters - pvalueCutoff = 0.01, qvalueCutoff = 0.05, pAdjustMethod = Benjamini–Hochberg correction and KEGG paramerters - pvalueCutoff = 0.05). Enriched KEGG terms were visualised using a custom R script. The network interaction between overrepresented GO BP pathways was visualized using the ClueGO application (v2.5.8)^99^ and its plugin CluePedia (v1.5.8)^100^ within the desktop version of the Cytoscape software (v3.8.2).^101^ The yFiles Organic Layout from the yFiles Layout Algorithms application (v1.1.1)^102^ was used to specify the design. Transcription factor enrichment analysis was performed using Enrichr^103^ and the ChEA3 database. The combined score was used to assess significance of enrichment. The SARS-CoV-2 gene set enrichment analysis was performed using Enrichr and the COVID-19 Drug and Gene Set Library.

#### Principal component analysis projection of circadian and SD transcript expression

To assess the circadian behaviour of the mouse lung we used principal component analysis (PCA). We first reduced the transcriptomic datasets to 10 circadian features, i.e., transcripts known to be highly rhythmic across murine organ systems (Arntl, Per2, Per3, Tef, Hlf, Dbp, Nr1d1, Nr1d2, Npas2, and Dtx4).^104^ The resultant transcript x sample matrices were log-transformed and then Z-score normalised column-wise to prepare the data for dimensionality reduction. Singular value decomposition was applied to the 16 samples collected at times ZT2, ZT8, ZT14, and ZT20 to obtain the principal directions (using the svd function in MATLAB v2020b). All lung samples (time course and SD) were then projected onto the 3D principal component space generated from the first three principal directions of the time course samples. The time point means of the projected time course samples were estimated by fitting Gaussian distributions. A shape-preserving cubic spline was fitted through the estimated means of the projected time course samples to approximate the expected circadian behaviour of the mouse lung (using the csape function in MATLAB). The Support Vector Machine approach (package gensvm v.0.1.5 in R v.4.1.1) with the linear kernel was then used to find the equation of the plane which optimally separated the control and SD lung samples in the 3D principal component space, and then all samples were projected onto the normal of the plane. A Wilcoxon’s rank sum test was carried out in MATLAB (ranksum function) for the projections on the normal to determine whether the null hypothesis that the control and SD samples belonged to the same population (same median) could be rejected.

### Quantification and statistical analysis

All data are expressed as mean + or ± SEM, and n represents the number of independent animals or replicates per group, as detailed in each figure legend. For comparisons between two groups only, a one-tailed unpaired Student’s t-test was used. Statistical significance of gene set overlaps was assessed by two-tailed Fisher’s exact test, assuming 21,647 total genes in the lung transcriptome as determined by the RNA-Seq data from SD and time series analysis in this study. Correlation between the qRT-PCR and RNA-Seq expression data was examined using two-tailed Pearson correlation analysis. Statistical testing was performed in R, MATLAB, and GraphPad Prism 9 (v9.1.2).

## Data Availability

All RNA-Seq data have been deposited on NCBI SRA and will be publicly available as of the date of publication. Accession numbers (Database: Bioproject PRJNA914246) are also listed in the key resources table. No original code was used in this study. Any additional information required to reanalyse the data reported in this paper is available from the lead contactupon request.

## Supplemental information

Document S1. Figures S1–S4

Table S1. RNA sequencing and differential gene expression analysis of Control and SD lung, related to Figure 1

Table S2. GO BP and KEGG enrichment analysis of SD differential genes, related to Figure 1

Table S3. Gene set enrichment analysis (GSEA) of SD differential genes using the COVID-19 Drug and Gene Set Library, related to Figure 3

Table S4. Overlapping gene/protein lists with our SD differential genes, related to Figure 3

Table S5. Transcription factor enrichment analysis of the SD significantly differential transcripts using Enrichr, related to Figure 1

Table S6. Metacycle analysis of lung time course RNA sequencing, related to Figure 2

Table S7. qPCR primer sequences
