# Supplementary material for: Sleep and circadian rhythm disruption alters the lung transcriptome to predispose to viral infection
Source: iScience. 2022 Dec 24;26(2):105877. doi: 10.1016/j.isci.2022.105877 (PMC9788990; doi:10.1016/j.isci.2022.105877)
Supplement: Document S1. Figures S1–S4 [file mmc1.pdf]

iScience, Volume *26*

Supplemental information

Sleep and circadian rhythm disruption alters the lung transcriptome to predispose to viral infection

Lewis Taylor, Felix Von Lendenfeld, Anna Ashton, Harshmeena Sanghani, Simona Di Pretoro, Laura Usselmann, Maria Veretennikova, Robert Dallmann, Jane A. McKeating, Sridhar Vasudevan, and Aarti Jagannath

Supplementary Figure 1

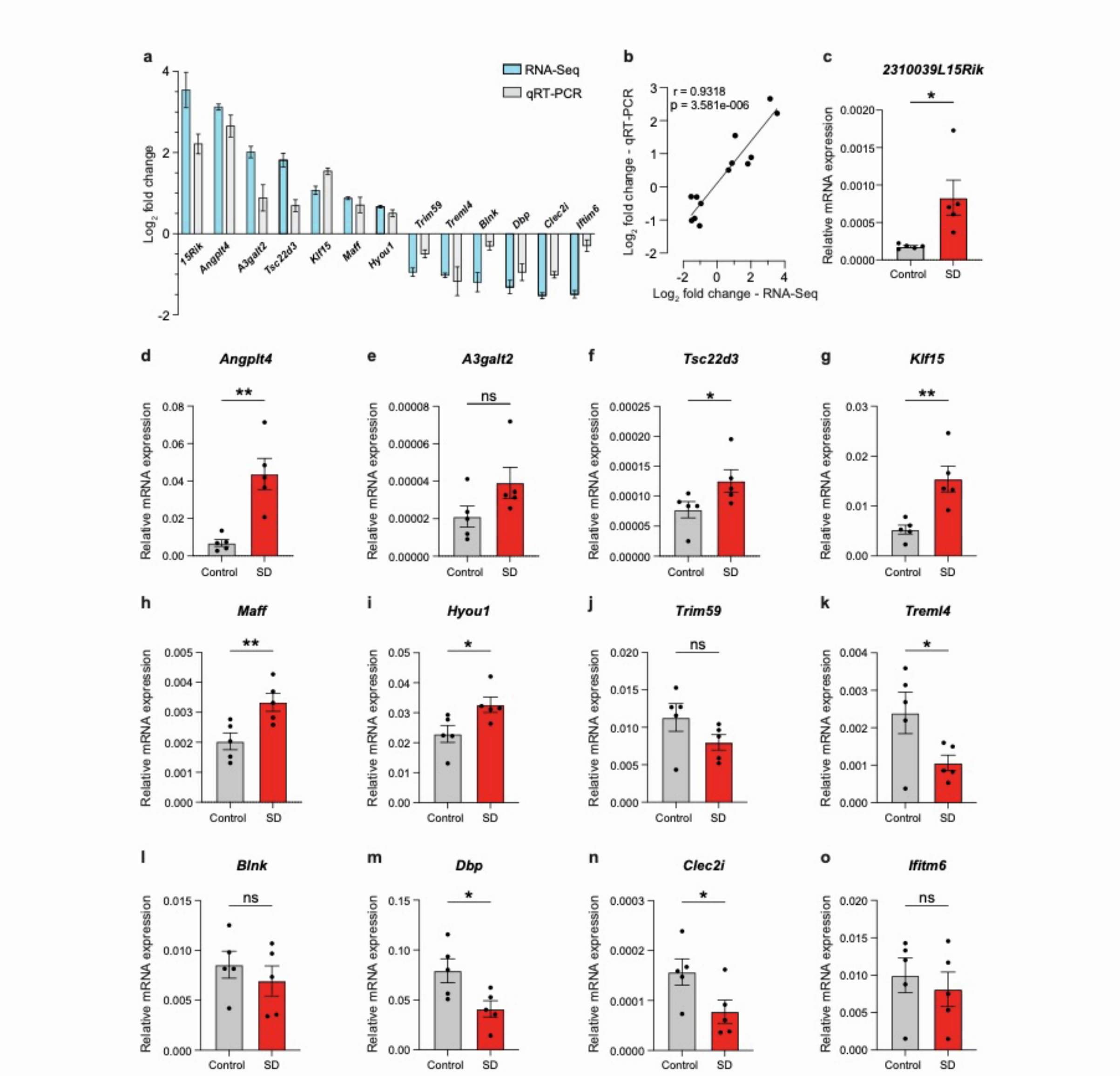

Supplementary Fig. 1 qRT-PCR analysis of independent, sleep deprived lung samples demonstrates the robustness of our RNA sequencing data. Related to Figure 1. Thirteen genes that has differential expression following SD in the lung were selected for qRT-PCR validation using independent control and SD lung samples. **a** Direct comparison of the Log_2 fold change detected for each gene by RNA-Seq and qRT-PCR. **b** Pearson correlation analysis between RNA-Seq and qRT-PCR gene expression data demonstrates these are significantly positively correlated. Relative expression of **c** *2310039L15Rik*, **d** *Angplt4*, **e** *A3galt2*, **f** *Tsc22d3*, **g** *Klf15*, **h** *Maff*, **i** *Hyou1*, **j** *Trim59*, **k** *Treml4*, **l** *Blnk*, **m** *Dbp*, **n** *Clec2i* and **o** *Ifitm6* in control and SD lung samples. For **b** data are mean, for **a** and **c-o** data are mean ± SEM. n=5. Statistical analysis was conducted by unpaired one-tailed student's t-test. ns $p > 0.05$, * $p < 0.05$, ** $p < 0.01$.

Supplementary Figure 2

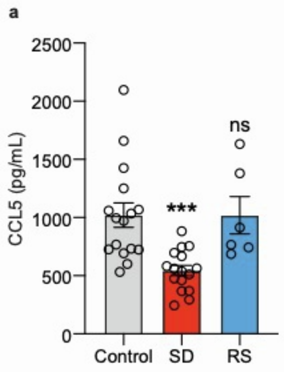

Supplementary Fig. 2 Three hours of recovery sleep reverses the suppressive impact of SD on CCL5 abundance in the lung. Related to Figure 1. a WT animals were allowed to sleep *ad libitum* (Control), sleep deprived (SD) between ZT0 – ZT6, or sleep deprived between ZT0 – ZT6 and then allowed to sleep *ad libitum* for three hours (RS). Lung tissue was collected and the level of CCL5 measured by ELISA. Data are mean ± SEM. n=6-16. Statistical analysis was conducted by one-way ANOVA with Dunnett's multiple comparisons correction. ns $p > 0.05$, *** $p < 0.001$.

Supplementary Figure 3

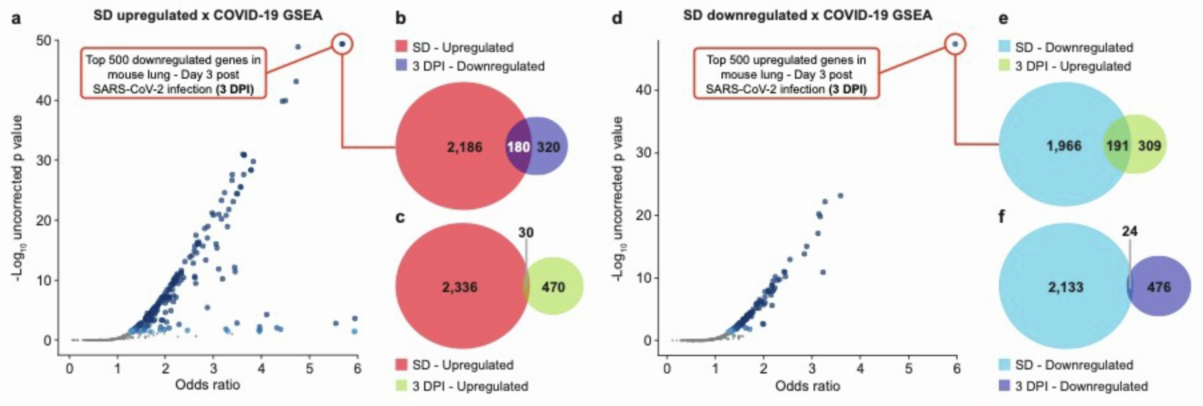

Supplementary Fig. 3 The transcriptome of the lung following SD is inversely correlated with the lung transcriptome during early-stage SARS-CoV-2 infection. Related to Figure 3. a Gene set enrichment analysis (GSEA) of SD upregulated genes using the COVID-19 Drug and Gene Set Library identified the most significantly enriched gene set as the top 500 genes downregulated in the mouse lung three days post SARS-CoV-2 infection (3 DPI), as determined by Li *et al.* 2021. There was a significant overlap between the SD upregulated genes and **b** the top 500 downregulated genes 3 DPI (Fisher's exact test p value = 7.7×10^{-26}), but not **c** the top 500 upregulated genes 3 DPI **d** GSEA of SD downregulated genes identified the most significantly enriched gene set as the top 500 genes upregulated 3 DPI There was a significant overlap between the SD downregulated genes and **e** the top 500 upregulated genes 3 DPI (Fisher's exact test p value = 8×10^{-37}), but not **f** the top 500 downregulated genes 3 DPI.

Supplementary Figure 4

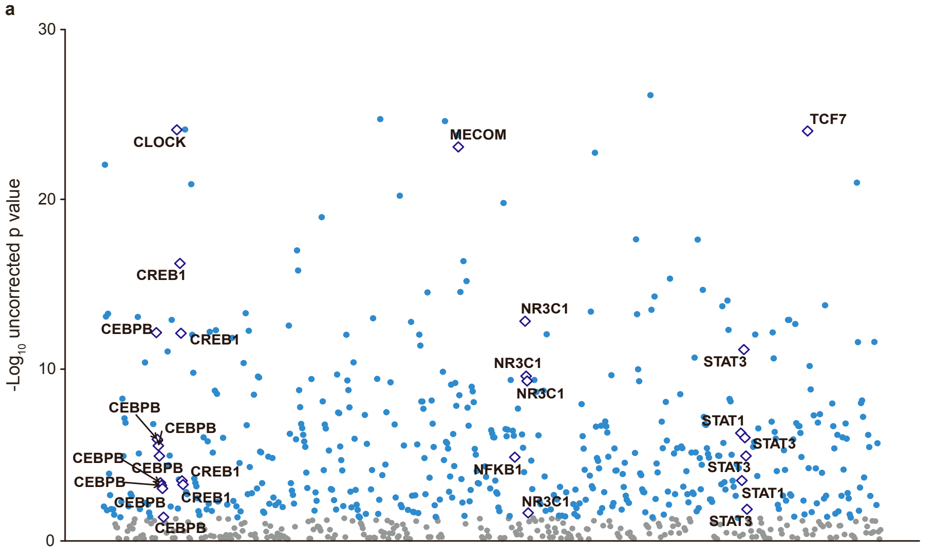

Supplementary Fig. 4 Transcription factor enrichment analysis of the significantly differential transcripts in the lung following acute SD. Related to Figure 1. The 4,523 SD differential genes were subjected to transcription factor enrichment analysis using the ChEA3 via the Enrichr online platform, with 457 individual transcription factor terms being significantly enriched (blue dots). Transcription factors of interest are highlighted (blue diamonds). p values are BH corrected, with an adjusted p value of < 0.05 considered significant. NR3C1 - Nuclear Receptor Subfamily 3 Group C Member 1 or Glucorticoid receptor highlighted – indicating less enrichment of this factor relative to others, such as CLOCK.

Supplementary Table 1. RNA sequencing and differential gene expression analysis of Control and SD lung. Related to Figure 1.

Supplementary Table 2. GO BP and KEGG enrichment analysis of SD differential genes. Related to Figure 1.

Supplementary Table 3. Gene set enrichment analysis (GSEA) of SD differential genes using the COVID-19 Drug and Gene Set Library. Related to Figure 3.

Supplementary Table 4. Overlapping gene/protein lists with our SD differential genes.Related to Figure 3.

Supplementary Table 5. Transcription factor enrichment analysis of the SD significantly differential transcripts using Enrichr. Related to Figure 1.

Supplementary Table 6. Metacycle analysis of lung time course RNA sequencing. Related to Figure 2.
